# Tubulin Acetylation Alone Does Not Affect Kinesin-1 Velocity and Run Length *In Vitro*

**DOI:** 10.1371/journal.pone.0042218

**Published:** 2012-08-01

**Authors:** Wilhelm J. Walter, Václav Beránek, Elisabeth Fischermeier, Stefan Diez

**Affiliations:** 1 B CUBE – Center for Molecular Bioengineering, Technische Universität Dresden, Dresden, Germany; 2 Max-Planck-Insitute of Molecular Cell Biology and Genetics, Dresden, Germany; The Hong Kong University of Science and Technology, Hong Kong

## Abstract

Kinesin-1 plays a major role in anterograde transport of intracellular cargo along microtubules. Currently, there is an ongoing debate of whether α-tubulin K40 acetylation directly enhances the velocity of kinesin-1 and its affinity to the microtubule track. We compared motor motility on microtubules reconstituted from acetylated and deacetylated tubulin. For both, single- and multi-motor *in vitro* motility assays, we demonstrate that tubulin acetylation alone does not affect kinesin-1 velocity and run length.

## Introduction

Kinesin-1 is a microtubule-based motor protein that converts the chemical energy derived from ATP hydrolysis into mechanical work to translocate processively towards the plus end of a microtubule. One of the multiple functions of kinesin-1 – and the first one that was discovered – is the transport of vesicles within neurons [1]. Thereby, kinesin-1 shows a preference for axonal microtubules over dendritic microtubules [2], [3] and experiments with truncated motor constructs have shown that the motor domain itself is sufficient to distinguish between the two kinds of microtubules [2]. This selectivity can be abolished by a mutation within the microtubule-binding surface of the kinesin-1 motor domain, indicating that track selection is an inherent property of the motor [4].

Acetylation of α-tubulin K40 is a well-known marker for highly posttranslationally modified, so-called ‘stable’, microtubules that account for the majority of the axonal microtubules [5]. Previous studies analyzed whether tubulin acetylation facilitates selective translocation of kinesin-1 *in vivo.* Cells were treated with trichostatin A (TSA) – an inhibitor of the histone deacetylase (HDAC) family – which subsequently caused an increase in overall tubulin acetylation [4], [6]. This led to an enhanced binding of kinesin-1 to the microtubules, a higher velocity, and a loss of the preference for axonal microtubules. Moreover, the addition of TSA to cells with impaired huntingtin protein - which causes a significant reduction of vesicle velocity and increases the frequency of waiting periods [7], [8] - restored velocity and frequency of vesicles back to wt levels [9]. More recently, however, two *in vivo* studies indicated that acetylation alone might not be sufficient to explain the preferential binding of kinesin-1 to axonal microtubules [10], [11].

The inconsistent results of the mentioned studies demonstrate the limitations of *in vivo* experiments as the complexity of the cellular environment often does not allow for definite conclusions. Especially the interpretation of results derived from experiments with chemical inhibitors requires caution, as other proteins besides tubulin might be affected. In order to analyze whether acetylation of the K40 residue alone is sufficient to modify kinesin-1 motility, we performed *in vitro* multi-motor gliding and single-motor stepping assays with microtubules reconstituted from acetylated and deacetylated porcine tubulin.

## Results and Discussion

**Figure 1:**
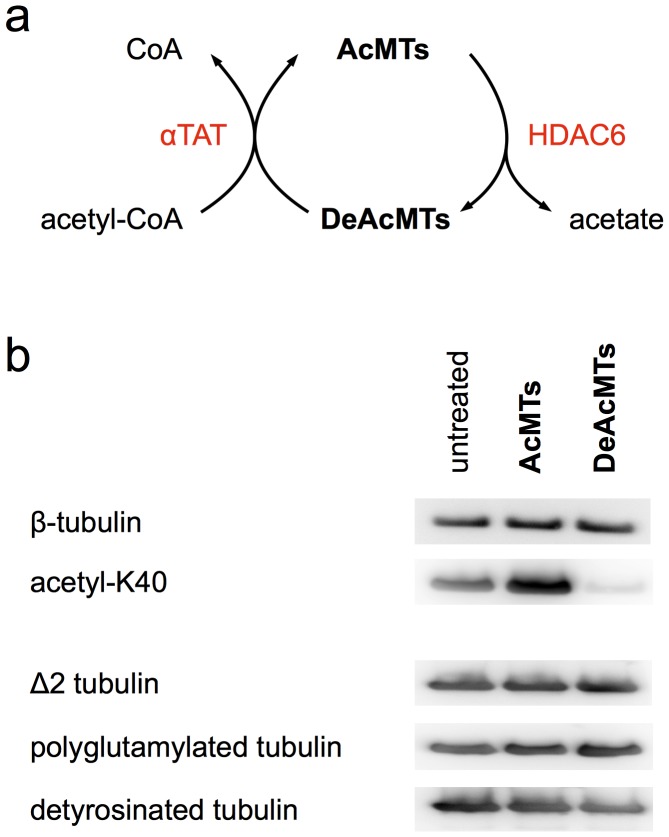
Tubulin acetylation and deacetylation. (**a**) Schematic representation of the acetylation and deacetylation of microtubules by αTAT and HDAC6, respectively. (**b**) Western blots of non-treated, acetylated, and deacetylated tubulin with anti-b-tubulin (SAP4G5, Abnova), anti-AcK40 (6–11B-1, Thermo Scientific), anti-detyrosinated-tubulin (AB3201, Millipore), anti-Δ2-tubulin (pab0202, covalab), and anti-polyglutamylated-tubulin (GT335, Enzo). αTAT-treated tubulin is highly acetylated, whereas HDAC6-treated tubulin is deacetylated. The levels of detyrosination, decarboxylation, and polyglutamylation are not affected by αTAT and HDAC6.

We prepared acetylated tubulin using mouse α-tubulin acetyltransferase (αTAT), which recently was discovered by two independent research groups to specifically acetylate α-tubulin K40 [12], [13] (Fig. 1a). Tubulin K40 deacetylation was performed by incubating tubulin with recombinant human histone transacetylase-like enzyme HDAC6, the role of which has been known for several years [14]. The success of acetylation and deacetylation was proven in a Western blot with antibodies specific for α-tubulin acetyl-K40 [15] (Fig. 1b). In order to rule out effects of HDAC6 or αTAT on other posttranslational tubulin modifications we additionally performed Western blots with antibodies against detyrosinated, decarboxylated (Δ2), and polyglutamylated tubulin. Neither of those modifications was affected (Fig. 1b).

**Figure 2:**
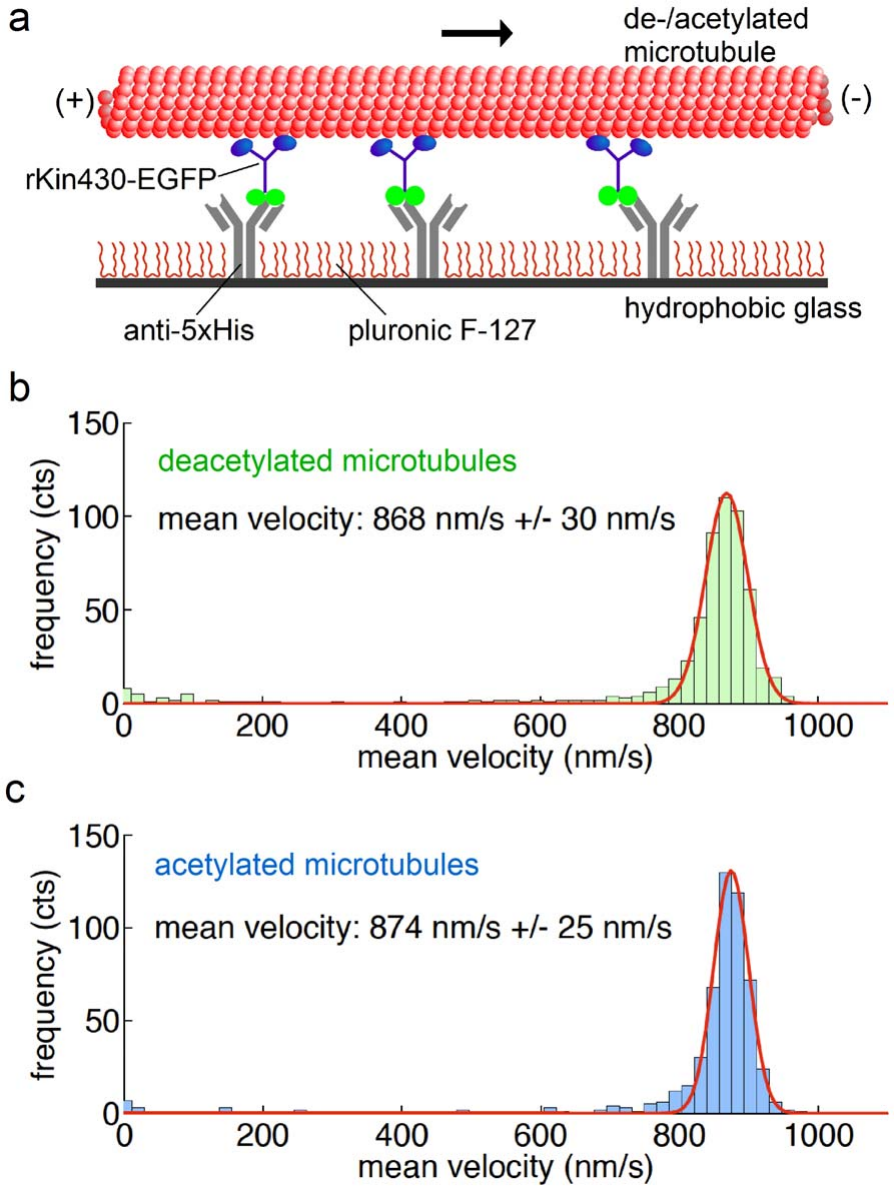
Tubulin acetylation does not affect microtubule velocity in kinesin-1 gliding assays. (**a**) Schematic representation of the experimental setup for multi-motor gliding assays. We measured the velocities of short rhodamine-labeled acetylated and deacetylated microtubules propelled by surface-bound motors in presence of 1 mM ATP. The mean gliding velocities of 568 deacetylated microtubules (**b**) and 532 acetylated microtubules (**c**) did not differ significantly. The errors represent the SD of the Gaussian distributions.

In multi-motor gliding assays [16] the velocities of rhodamine-labeled acetylated and deacetylated microtubules propelled by surface-bound, truncated rat kinesin-1 labeled by EGFP (rKin430-EGFP) [17] were determined in the presence of 1 mM ATP (Fig. 2a). We determined gliding velocities of 868+/−30 nm·s-1 (mean +/− SD, n = 568 microtubules) and 874+/−25 nm·s-1 (n = 532) for deacetylated and acetylated tubulin, respectively (Fig. 2b and c). The 0.7% difference in the mean velocities is statistically significant (p = 0.0003) due to the high number of observes microtubules. However, on the other hand this difference is equivalent to the effect of an increase in temperature by ΔT = 0.1 K (see Methods). As we are experimentally able to control the temperature only within a range of +/−0.5 K, we consider the observed velocity difference to be not significant.

**Figure 3:**
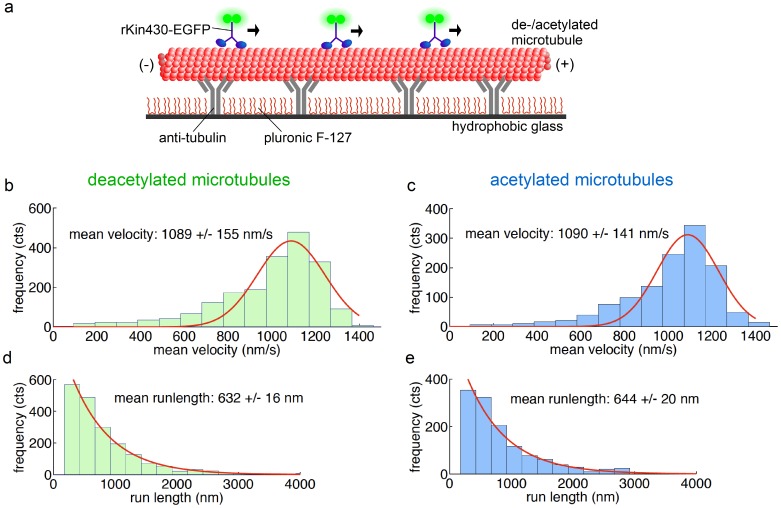
Tubulin acetylation does not affect the kinetics of kinesin-1 stepping on microtubules. (**a**) Schematic representation of the experimental setup for single-motor stepping assays. Single rKin430-EGFP motors walk on a microtubule attached to the surface via anti-tubulin antibodies. By tracking the positions of the motors we determined the mean velocities and mean run lengths for deacetylated and acetylated microtubules. The mean velocities of (**b**) 1977 motors on deacetylated and (**c**) 1283 motors on acetylated microtubules did not differ significantly. The errors represent the SD of the Gaussian distributions. Mean run lengths of (**d**) 1949 single motors on deacetylated and (**e**) 1270 motors on acetylated microtubules were determined by fitting the cumulative probability distributions. The run lengths on deacetylated and acetylated microtubules did not differ significantly. The errors were estimated with the bootstrap technique.

In single-motor stepping assays [18] the velocities of individual rKin430-EGFP motor molecules on acetylated and deacetylated microtubules attached to a glass surface were determined in the presence of 1 mM ATP (Fig. 3a). We determined stepping velocities of 1089+/−155 nm·s^−1^ (*n* = 1977 motors) and 1090+/−141 nm·s^−1^ (*n* = 1283) on deacetylated and acetylated microtubules, respectively (Fig. 3b and c). Again, these values did not differ significantly (p = 0.85). Moreover, independent of the acetylation status, the run lengths (632+/−16 nm, *n* = 1949, on deacetylated and 644+/−20 nm, *n* = 1270, on acetylated microtubules, measured at a modest laser illumination intensity) did not differ significantly (p = 0.44) (Fig. 3d and 3e).

In both, multi-motor gliding and single-motor stepping assays with microtubules reconstituted from acetylated and deacetylated tubulin we did not observe a direct effect of K40 acetylation on kinesin-1 velocity or run length. We thus hypothesize that the differences in motor protein motility previously observed *in vivo* [4], [6], [9] might simply correlate with - but not be induced by – microtubule acetylation. On one hand, acetylation often coexists with other posttranslational modifications. *in vivo* experiments indicate that detyrosination of α-tubulin in axonal microtubules might be responsible for the selective translocation of kinesin-1 [4]. Also, chemically inhibited tubulin acetylation *in vivo* may have effected the acetylation of additional putative acetylation sites in the tubulin dimer [19] or the acetylation of other proteins but tubulin. On the other hand, posttranslational modifications are not the only microtubule features that potentially interfere with molecular motors. Within the cell microtubules display a range of accessory proteins, so called microtubule-associated proteins (MAPs). Although the K40 acetylation site is located in the inner lumen of the microtubule [20], rendering a direct electrostatic effect on MAPs interacting with the outer surface of the microtubule unlikely, small conformational changes induced by K40 acetylation may allosterically propagate to the respective MAP binding sites. Previous results might thus be secondary effects provoked by the interaction of kinesin-1 with MAPs that potentially recognize tubulin acetylation. In particular, the protein tau, which has been described to interfere with kinesin-1 stepping [21] and tubulin acetylation [22], might play a significant role.

In summary, by performing single- and multi-motor *in vitro* motility assays, we found that tubulin acetylation alone does not affect kinesin-1 velocity and run length. Rather than the acetylation state of microtubules as such, its combination with additional posttranslational modifications or MAPs, may thus be responsible for guiding kinesin-1 towards axonal transport *in vivo*. Further experiments – including *in vitro* measurements with controlled populations of posttranslationally modified microtubules and additional binding partners – will be required to fully understand the selective translocation of kinesin-1.

## Materials and Methods

### Tubulin Acetylation and Deacetylation

Murine αTAT was recombinantly expressed in the *E. coli* strain BL21 as a GST fusion protein and purified as described previously [13]. The αTAT expression plasmid was a generous gift by Prof. Jacek Gaertig (University of Georgia, Georgia, USA). 25 µM porcine tubulin was acetylated by ∼3 mM αTAT (50 mM Tris-HCl, 50 mM PIPES, 0.5 mM acetyl-CoA, pH 7.6) or deacetylated by 50 µM recombinant human HDAC6 (Enzo Life Sciences, USA) (50 mM Tris-HCl, 50 mM PIPES, pH 7.6) for 90 min at 28°C. Microtubules were polymerized for 90 min at 37°C in presence of 1.25 mM GMPCPP and 1.25 mM MgCl_2_. Polymerized microtubules were pelleted at 200,000 g for 10 min and resuspended in BRB80 (80 mM PIPES, 1 mM MgCl_2_, 1 mM EGTA, pH 6.9) with 10 µM taxol.

### Gliding and Stepping Motility Assays

All experiments were performed with truncated, EGFP-labeled kinesin-1 constructs (rKin430-EGFP), which contained the first 430 aa of kinesin-1 fused to a EGFP and a His tag at the tail domain [15]. Multi-motor gliding motility assays and imaging were performed at room temperature (22–23°C) as described previously [23] except for the final assay solution (35 mM PIPES, 1 mM MgCl_2_, 1 mM EGTA, 20 mM KCl, 1 mM ATP, 0.2 mg/ml catalase, 0.1 mg/ml glucose oxidase, 40 mM D-glucose, 10 mM DTT, 10 µM taxol, pH 7.4). Single-motor stepping assays and imaging at modest laser illumination intensity were performed as described previously [24] under the same buffer conditions as the gliding assays.

### Data Analysis

Velocities of microtubules and single motors, as well as the run lengths of single motors were obtained using FIESTA tracking software [25]. Mean velocities were determined by fitting the velocity histograms to Gaussian functions. In the analysis of the stepping assays only motor molecules moving over longer distances than 200 nm along the microtubule axis were considered. The significance of velocity differences was tested using t-test statistics. The temperature change ΔT = 0.1 K required to increase the mean velocity from v_1_ = 868 nm·s^−1^ to v_2_ = 868 nm·s^−1^ was estimated assuming a two-fold velocity increase every 10 K [26] using: ΔT = 10 K · log(v_2_/v_1_)/log(2) derived from: v_2_ = v_1_·2̂(ΔT/10 K).

To obtain bin-size independent values of the mean run lengths we analyzed the cumulative probability distributions [27]. These distributions were fitted with functions 1 - exp[(*x*_0_−*x*)/*t*], where the only fitted parameter *t* is the mean run length of the distribution and *x*_0_ the lower limit for runs included into the analysis. As an error estimate for the mean run length, we considered 200 bootstrap samples [28] and fitted them as described above. Standard deviations of the sampled sets were used as error estimates. The significance of the differences in the run lengths was tested using Kolmogorov–Smirnov statistics [29]. Repeating the run lengths measurements at 40% and 80% increased laser intensities allowed us to extrapolate to run lengths free of photobleaching effects [30]. For both, deacetylated and acetylated microtubules, we found that these photobleach-free run lengths (though approximately 8% longer than the values given in the main text) did not differ significantly. All fitting was performed in MATLAB (MathWorks, USA).
